# Performance of the modified HEART score in an Asian population

**DOI:** 10.1186/s12245-020-00300-1

**Published:** 2020-08-19

**Authors:** Shanaz Matthew Sajeed, Michael P. De Dios, Dan Wei Jun Ong, Amila Clarence Punyadasa

**Affiliations:** 1 Department of Emergency Medicine and Department of Intensive Care Medicine, Ng Teng Fong General Hospital, 1 Jurong East Street 21, Singapore, 609606 Singapore; 2 Department of Emergency Medicine, Ng Teng Fong General Hospital, 1 Jurong East Street 21, Singapore, 609606 Singapore; 3 Department of Respiratory Therapy, Ng Teng Fong General Hospital, 1 Jurong East Street 21, Singapore, 609606 Singapore

**Keywords:** Chest pain, Acute coronary syndrome, Emergency department, Major adverse cardiac event, Modified HEART score

## Abstract

**Introduction:**

Chest pain is the most common potentially life-threatening presentation to the emergency department (ED). Furthermore, the identification of acute coronary syndrome (ACS) including its risk stratification and subsequent disposition can be challenging. The original HEART score was derived as a predictive tool to risk stratify patients presenting with undifferentiated chest pain (CP) and aid physician decision-making. However, it utilized conventional troponins as its cardiac biomarker component. Our study aims to assess the utility of the modified HEART score with highly sensitive troponins in an Asian setting with mixed ethnicity to determine if it corroborates the findings of another recent Chinese study by Chun-Peng et al. (Journal of Geriatric Cardiology 13:64–69, 2016).

**Methods:**

Clinical data from 413 patients presenting to the ED for evaluation of chest pain were analyzed. The predictive value of the modified HEART score for determining major adverse cardiac events (MACE) was then evaluated.

**Results:**

A total of 49 patients (11.9%) had a MACE: 31 patients (7.5%) underwent PCI and 1 patient (0.2%) underwent CABG. There were 17 (4.1%) deaths.

Three risk groups were elucidated based on MACE. In the low-risk group (0–2), there were 72 patients (17.4%), with a MACE rate of 1.4%. In the intermediate-risk group (3–5), there were 233 patients (56.4%), with a MACE rate of 5.2%. In the high-risk group (6–10), there were 108 patients (26.2%), with a MACE rate of 33.3%.

**Conclusion:**

The modified HEART score is an effective risk stratification tool in an ethnically diverse Asian population. Furthermore, it identifies low-risk patients who are candidates for early discharge from a local emergency department.

## Introduction

Amongst patients who present to the emergency department (ED) with chest pain, the difficulty lies in distinguishing cardiac from non-cardiac chest pain. Patients may not present with typical symptoms. Furthermore, there are conditions that may mimic an acute coronary syndrome (ACS). This often presents the clinician with a diagnostic dilemma, and in such situations, the safest option remains to admit the patient for further investigations and observation. Making an accurate diagnosis in the ED is important as an inappropriate discharge may have life-threatening consequences. Conversely, unnecessary admissions would result in increased utilization of health care resources and increased cost [1].

The diagnosis of ACS is based on history, ECG findings, and biochemical markers such as troponins. Elements of these have been incorporated into numerous scoring systems. Scoring systems such as the Thrombolysis in Myocardial Infarction (TIMI) and Global Registry of Acute Coronary Events (GRACE) are commonly used. However, their utility in discriminating cardiac from non-cardiac causes of chest pain is poor [2, 3]. They are used to risk stratify patients who already have proven ACS [4]. The challenge, however, remains for the emergency physician to diagnose ACS.

The HEART score had been developed to help emergency physicians risk stratify patients presenting with chest pain and determine their likelihood of developing a major adverse cardiac event (MACE) within 6 weeks [5]. While it does not discriminate between cardiac and non-cardiac chest pain, its primary role is to act as a decision-making tool to help identify low-risk patients in those with suspected ACS. It can be done at the bedside and has been prospectively validated by Backus et al. in 2013. When compared to the GRACE and TIMI score, the HEART score was found to be more accurate in predicting outcome [6]. The elements of the HEART score include the classical considerations for risk stratification: history, ECG, age, risk factors, and troponin (HEART). Each can be scored with zero, one, or two points, depending on the extent of the abnormality. The HEART score is the sum of these five elements.

The modified HEART score (see Table 1) was introduced to incorporate the use of highly sensitive troponins and was retrospectively validated in a recent Chinese study [7]. It may complement MACE risk assessment and aid in decision-making for patients presenting to the emergency department with suspected ACS [6].

**Table 1:** Modified HEART risk score for chest pain patients

Components	Rank	Points
History	Highly suspicious	2 points
	Moderately suspicious	1 point
	Slightly or non-suspicious	0 points
ECG	Significant ST depression	2 points
	Nonspecific repolarization	1 point
	Normal	0 points
Age	≥ 65 years	2 points
	> 45 years and < 65 years	1 point
	≤ 45 years	0 points
Risk factors	≥ 3 risk factors* or history of atherosclerotic disease^	2 points
	1 or 2 risk factors	1 point
	No risk factors	0 points
Troponin	≥ 3 times of normal limit	2 points
	> 1 to < 3 times of normal limit	1 point
	Within normal limit	0 points
Range		0–10 points

*Risk factors: diagnosed hypertension, diagnosed hypercholesterolemia, diagnosed diabetes mellitus, family history of premature coronary artery disease, current smoking (< 1 month), and obesity (body mass index 30 kg/m^2^)

^History of atherosclerotic disease includes myocardial infarction, percutaneous intervention, coronary artery bypass graft, ischemic stroke, peripheral arterial disease, or carotid artery disease

The sensitivity for detecting conventional cardiac troponin T and I approach 100% when sampled 6–12 h after acute chest pain onset [8]. Recent advances in technology have resulted in more sensitive and precise assays, able to detect circulating Tn levels more precisely than conventional ones, particularly in the low range. These have been termed high-sensitivity cardiac troponins (hs-cTn). Most hospitals now have replaced conventional cTn tests in the last couple of years with the new 5th generation hs-cTn T and I assay which can detect troponin at concentrations 10- to 100-fold lower than conventional assays.

These hs-cTn have a higher diagnostic accuracy which provides earlier detection of acute myocardial infarction (AMI) [9, 10]. The negative predictive value (NPV) of hs-cTn assays is > 95% for AMI exclusion when patients are tested on arrival at the ED [11]. If this is repeated at 3 h, this rises to nearly 100% [12]. Furthermore, hs-cTn are capable of identifying higher-risk patients in the conventional Tn (cTn) negative group [13–15], thereby having important implications in driving decision-making during initial ACS management.

The original study by Six and Backus et al. stratified patients into low, intermediate, and high risk based on the HEART score [16, 17]. The results revealed that a low HEART score (0–3) conferred a 1.7% MACE rate, an intermediate HEART score (4–6) a 16.6% MACE rate, and a high HEART score (7–10) a 50.1% MACE rate. In the study by Chun-Peng et al. using the modified HEART score, low-risk patients were classified as having a score of 0–2 which differed from the original HEART score [7]. The reported MACE rate for this new low-risk category was reported as 1.1%. The MACE rate increased significantly in the intermediate-risk group (score 3–4) to 18.5%.

Our study aims to assess the utility of the modified HEART score with highly sensitive troponins in an Asian setting to determine if it corroborates the findings of the recent study by Chun-Peng et al. [7].

One of the criticisms of the validation study of the original HEART score by Backus et al. [17] is that each emergency department used different cutoff values for the troponin. However, none of the emergency departments was reported to have used highly sensitive troponins apart from one recent publication assessing the use of highly sensitive troponin in the modified HEART score [18]. We aim to retrospectively analyze patients that come into our emergency department with chest pain and calculate the modified HEART score. A similar endpoint of 6 weeks major adverse cardiac events (MACE) will be used which is comparable to the original study. The MACE looked at are all-cause mortality, myocardial infarction, or coronary revascularization.

## Methods

### Study population

**Fig. 1:**
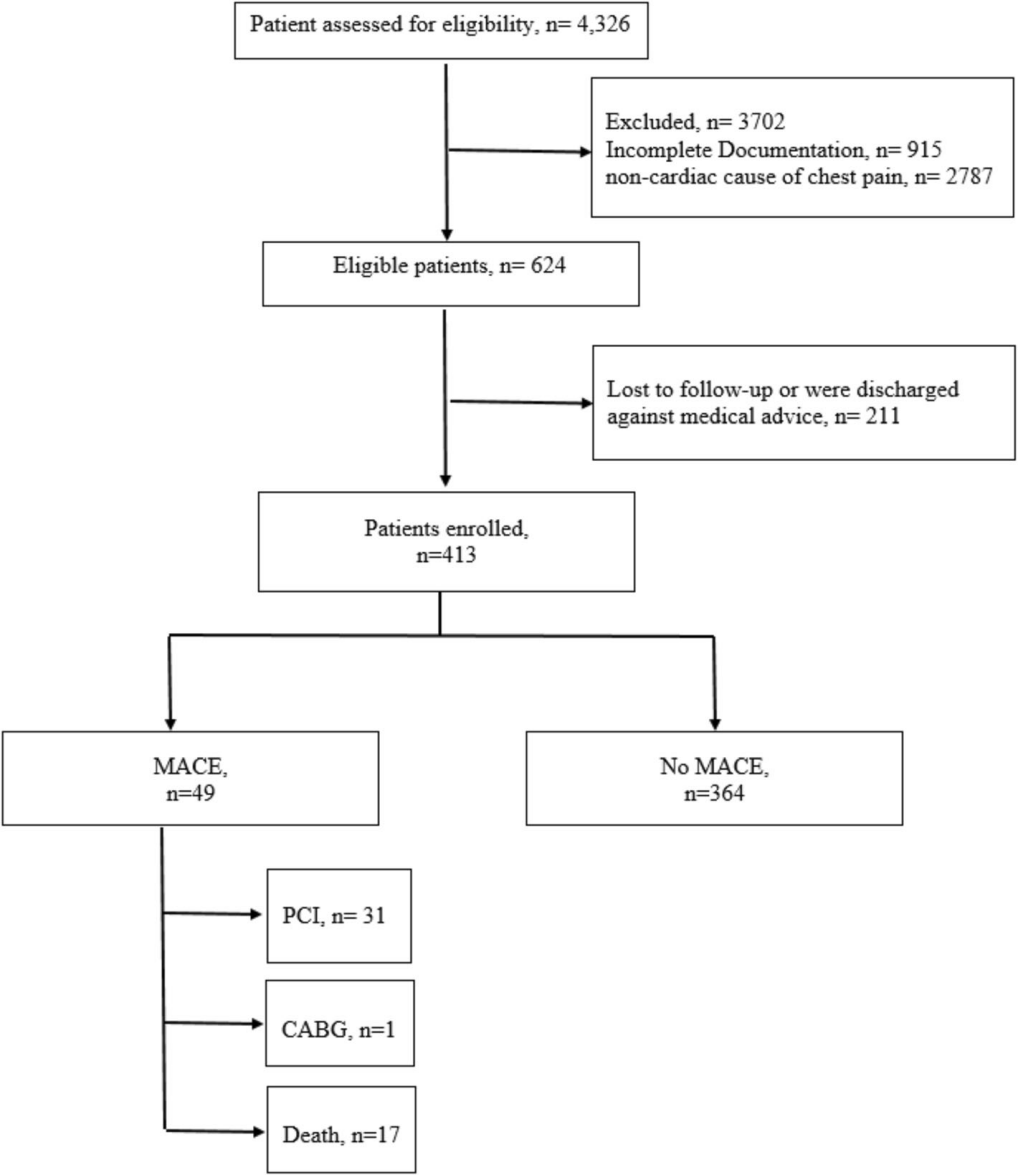
Flow chart of study participants. ACS: acute coronary syndrome; AMI: acute myocardial infraction; CABG: coronary artery bypass graft; MACE: major adverse coronary events; PCI: percutaneous coronary intervention

This was a retrospective cohort study performed at a 700-bed general hospital in Singapore with an annual ED attendance of 110,300 patients. All adult patients above the age of 18 who attended the emergency department from the period of January 2016 to June 2016 with chest pain were included in the study. Patients with STEMI were excluded from the study. All admission and follow-up data were retrieved from the hospital records. The patients were selected consecutively during the study period (see Fig. 1). Setting the acceptable significance level (*α* = 0.05) for 2-tailed alternative hypotheses and assigning the power of the study at 80% (*β* = 0.2), we estimated the sample size to be 407 for this study. Ethics approval from the National Health Group Domain Specific Review Board was obtained for the collection and analysis of data.

### Modified HEART score

In the modified HEART risk score, the “troponin” component was highly sensitive troponin I instead of the conventional troponin. The standard cutoff value of the 99th percentile of troponin was used in determining normal and abnormal values. The other components of the HEART score remained the same as those in the conventional score. The history was based on the narrative of the electronic charts and categorized into highly suspicious, moderately suspicious, or slightly or non-suspicious based on the index of suspicion of the reviewer based on elements such as the onset, nature, and duration of the pain as well as response to nitrates. Charts with incomplete history or poor documentation were excluded. The ECG was read by an independent consultant cardiologist not involved in data analysis of the study. The modified HEART score is shown in Table 1.

### Outcome measure

The outcome measure was the occurrence of MACE. MACE was defined as a composite of acute myocardial infarction (AMI), percutaneous intervention (PCI), coronary artery bypass graft (CABG), or all-cause death, within 6 weeks after initial presentation.

### Statistical analysis

Statistical analysis was performed using the SPSS statistical package (version 23.0, SPSS Inc., Chicago, IL, USA). The continuous variable was presented as mean ± SD. Categorical variables were given as frequencies and percentages. The discriminative power of the score was evaluated using the C statistic, which is the area under a receiver operating characteristic (ROC) curve for binary outcomes. Differences among groups were assessed by means of nonparametric test. *χ*^2^ test was used to evaluate differences in the event rates for increasing risk score. Student’s *t* test was used to compare differences between 2 groups for continuous quantitative variables.

## Results

**Fig. 2:**
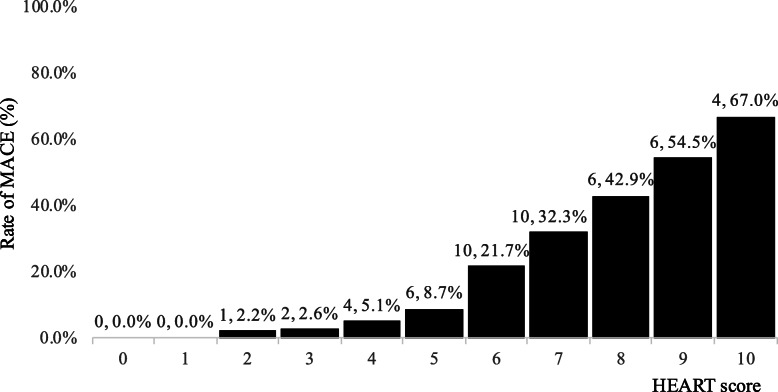
Rate of MACE (*n*, %). According to the HEART score (HEART: history, electrocardiograph (ECG), age, risk factors, and troponin; MACE: major adverse cardiac events), MACE increased significantly as the risk score increased (*P* < 0.001 by *χ*^2^ for trend)

The study population was derived from 413 patients with chest pain presenting to the ED for evaluation (see Fig. 1). A total of 49 patients (11.9%) had a MACE: 31 patients (7.5%) underwent PCI and 1 patient (0.2%) underwent CABG. There were 17 (4.1%) deaths. The baseline characteristics of the study cohort are shown in Table 2. The C statistic for the score in the whole study group was 0.83 (95% CI 0.77 to 0.89). There was a progressive, significant pattern of increasing event rates as the score increased in the study cohort (*P* < 0.001 by *χ*^2^ for trend; Fig. 2).

**Table 2:** Baseline characteristics risk factors of the study cohort

Age, years	59.77 ± 16.92
Male	248 (60.1%)
History of atherosclerotic disease	120 (29.1%)
Hypertension	222 (53.8%)
Hyperlipidemia	166 (40.2%)
Diabetes mellitus	144 (34.9%)
Obesity (BMI > 30)	0 (0.0%)
Smoking	60 (14.5%)
Positive family history	20 (4.8%)
Ethnicity	
Chinese	227 (55.0%)
Malay	109 (26.4%)
Indian	50 (12.1%)
Others	27 (6.5%)

The rate of MACE in the three groups was different (*P* < 0.001 by *χ*^2^ test). Data are mean ± SD or *n* (%).

*CABG* coronary artery bypass graft, *MACE* major adverse cardiac events, *PCI* percutaneous coronary intervention

To stratify chest pain patients in the ED, patients were classified into three groups (Table 3). The boundaries of low-, intermediate-, and high-risk groups were defined as having a MACE rate of ≤ 2.5%, > 2.5% but ≤ 20%, and > 20% respectively. In the low-risk group, there were 72 patients (17.4%) out of which 1 patient underwent PCI. The MACE rate in this group was 1.4%. In the intermediate-risk group, there were 233 patients (56.4%) out of which there were 6 deaths and 6 PCIs. The MACE rate was 5.2% in this group. In the high-risk group, there were 108 patients (26.2%) out of which there were 24 PCIs, 11 deaths, and 1 CABG. The MACE rate in this group was 33.3% (Table 3).

**Table 3:** Classification of patients

Classification	Score	Patients, *n* (%)	MACE (*n*)	Rate of MACE (%)*
Low risk	0–2	72 (17.4%)	PCI (1)	1.4
Intermediate risk	3–5	233 (56.4%)	PCI (6), death (6)	5.2
High risk	6–10	108 (26.2%)	CABG (1), PCI (24), death (11)	33.3

*CABG* coronary artery bypass graft, *MACE* major adverse cardiac events, *PCI* percutaneous coronary intervention

*The rate of MACE in the three groups was different (*P* < 0.001 by *χ*^2^ test)

The rationale for choosing classification of HEART score 0–2 (low risk), 3–5 (intermediate risk), and 6–10 (high risk) is because there was a significant difference in the trend of MACE between the three groups using this classification (low risk, intermediate risk, and high risk), *P* < 0.001 by *χ*^2^ test. The MACE rate rose to 2.6% with a HEART score of 3, above the threshold of 2.5% which we would consider low risk. Between a HEART score of 5 and 6, the HEART score increased from 8.7 to 21.7% defining the boundary between intermediate and high risk (see Fig. 2). This risk group classification differs from the original HEART score but is somewhat similar to the validation study for the modified HEART score by Chun-Peng et al. [7].

The numerical distribution of the score’s five components in the groups with or without MACE is shown in Table 4. Amongst the components of the HEART score that predict MACE, history, ECG, risk factors, and troponin reached statistical significance (*P* < 0.05 by *χ*^2^ for trend). In our study, the MACE rate was 3.1% (9/294) if the troponin was within the normal range (score 0 points for troponin component), the MACE rate was 22.7% (10/44) if the troponin score was 1 point, and the MACE rate was 40.0% (30/75) if the troponin score was 2 points. There was a progressive, significant pattern of increasing event rates as the troponin score increased (*P* < 0.001 by *χ*^2^ for trend).

**Table 4:** Number of patients in each component of the modified HEART score

	No MACE, *n* = 364			MACE, *n* = 49			*P* value for trend
	0	1	2	0	1	2	
History*	105 (28.8%)	149 (40.9%)	110 (30.2%)	7 (14.3%)	22 (44.9%)	20 (40.8%)	0.031
ECG**	255 (70.1%)	95 (26.1%)	14 (3.8%)	12 (24.5%)	15 (30.6%)	22 (44.9%)	< 0.001
Age	77 (21.2%)	150 (41.2%)	137 (37.6%)	5 (10.2%)	24 (49.0%)	20 (40.8%)	0.209
Risk factors*	85 (23.4%)	133 (36.5%)	146 (40.1%)	5 (10.2%)	17 (34.7%)	27 (55.1%)	0.017
Troponin**	285 (78.3%)	34 (9.3%)	45 (12.4%)	9 (18.4%)	10 (20.4%)	30 (61.2%)	< 0.001

Data are *n* (%)

*HEART* history, ECG, age, risk factors, and troponin; *MACE* major adverse cardiac events

**P value < 0.01; *P value < 0.05

The mean HEART score was 6.65 ± 1.97 in the MACE group and 4.02 ± 1.87 in the non-MACE group (*P* < 0.001 by *χ*^2^ test).

In our study, we found the number of risk factors had significantly affect MACE (*P* = 0.020). However, we found certain risk factors such as diabetes mellitus and smoking increase MACE by 1.638 and 1.383 respectively, but it is not statistically significant (refer to Table 5).

**Table 5:** Risk assessment of risk factors that will result in MACE

Risk factors	Odds ratio	*P* value	95% confidence interval
History of atherosclerotic disease	0.439	0.060	0.186–1.035
Hypertension	0.401	0.027	0.178–0.902
Hyperlipidemia	0.360	0.008	0.169–0.765
Diabetes mellitus*	1.638	0.236	0.724–3.708
Smoking*	1.383	0.485	0.556–3.435
Positive family history	0.102	0.052	0.010–1.022
Age	0.402	0.037	0.171–0.945
Male	0.400	0.001	0.230–0.697
Intermediate HEART score	0.219	< 0.001	0.104–0.457
High HEART score**	5.893	0.002	1.940–17.899

*MACE* major adverse cardiac events

**Significantly increase odds of MACE; *P* value < 0.05

*Increase odds of MACE but not significant

## Discussion

In line with previous published literature, we classified our patients into low-, intermediate-, and high-risk groups according to the MACE rate to explore the potential usefulness of the score in our patient population. There appears to be variation in the boundaries for low-, intermediate-, and high-risk patients [16, 18–22].

In our study, the boundaries of low-, intermediate-, and high-risk groups were defined as having a MACE rate of ≤ 2.5%, > 2.5% but ≤ 20%, and > 20% respectively. In our study, the score ranges in the three risk groups were 0–2 points (low risk), 3–5 points (intermediate risk), and 6–10 points (high risk), respectively. This varies from other previous studies that did not use highly sensitive troponins but is largely consistent with studies that used the modified HEART score with highly sensitive troponins [7, 23], except for a modification of the boundary for the low-risk group. In the study by Chun Peng et al. [7], the authors used a lower boundary of 5% MACE rate to define low risk. We feel that it would be unsafe to discharge a patient with a 6-week MACE of 4.9%. As such, we have considered a MACE of anything less than 2.5% to be low risk.

In the low-risk group, the MACE rate was only 1.4%, which is only marginally higher than the 1.1% reported by Chun Peng et al., and the only event was PCI. Patients in this group could be discharged safely from the emergency department [24]. In the intermediate-risk group, the MACE rate was 5.2%, which is lower than that found in previous studies [7, 17, 18]. A MACE rate of 5.2% is still unacceptably high to consider a discharge, and patients in this group should likely still be admitted for clinical observation and further evaluation. In the high-risk group, the MACE rate increased to 33.3% and, although lower than that found in other studies, was a significant increase from the intermediate-risk group (*P* value < 0.001). Patients in this group should be admitted to the hospital and should be considered for early intervention. The lower than expected MACE for these high-risk patients could be due a number of reasons including a varied patient population comprising different ethnicities from most previously described studies, differences in disease prevalence, and subjectivity to the history component of the scoring system as well as a relatively low sample size.

Unsurprisingly, troponin rise was an important risk factor and a good predictor of MACE with a progressive significant pattern of increased MACE rate with a rising troponin level. At present, the HEART score gives equal weightage to the risk factors. However, we found that certain risk factors such as diabetes mellitus significantly increase MACE more so than other risk factors.

Limitations of our study include the fact that our sample size is relatively small, and it is a retrospective analysis rather than a large-scale multicenter prospective validation study. Although the previously chosen weights of the 5 components of the HEART score have been supported by multivariable statistical analysis [7, 16, 19, 23], more study is needed to determine if diabetes mellitus and other risk factors will result in a higher probability of MACE. This will help to give appropriate weightages to different risk factors and help to improve the HEART scoring system. However, at this juncture, this is simply conjecture and a prospective study is warranted to further evaluate the relative effects of the risk factors as well as the use of the modified HEART score. We also recognize the limitations of classification of history to various degrees of suspiciousness for ACS based on retrospective chart analysis. There may be an element of subjectivity in both the history and ECG interpretation which may lead to misclassification bias. Also, while we aimed to compare our results with the large Chinese validation study of the modified HEART score by Chun Peng et al. [7], we do recognize that our population is more heterogeneous compared to the Chinese study. However, that being said, 55% of the participants were ethnically Chinese while the vast majority were Asian (Table 2).

In conclusion, our study supports the current practice of utilizing the modified HEART score to identify low-risk patients for early discharge from the emergency department in the local population. This can be combined into a HEART pathway which involves an accelerated protocol that measures 2 serial troponins 3 h apart [25–27]. This strategy may further decrease the MACE miss rate.

## Data Availability

The datasets used and/or analyzed during the current study are available from the corresponding author on reasonable request.

## Abbreviations

ED: Emergency department
ACS: Acute coronary syndrome
TIMI: Thrombolysis in Myocardial Infarction
GRACE: Global Registry of Acute Coronary Events
MACE: Major adverse cardiac events
HEART: History, ECG, age, risk factors, and troponin
hs-cTn: Highly sensitive troponin
AMI: Acute myocardial Infarction
PCI: Percutaneous intervention
CABG: Coronary artery bypass graft
